# Effect and Optimal Level of Dietary Dried Watermeal (*Wolffia globosa*) Supplementation on the Production Performance of Two-Spotted Crickets (*Gryllus bimaculatus*)

**DOI:** 10.3390/ani15142052

**Published:** 2025-07-11

**Authors:** Jamlong Mitchaothai, Nils T. Grabowski, Rachakris Lertpatarakomol, Tassanee Trairatapiwan, Achara Lukkananukool

**Affiliations:** 1 Office of Administrative Interdisciplinary Program on Agricultural Technology, School of Agricultural Technology, King Mongkut’s Institute of Technology Ladkrabang (KMITL), Bangkok 10520, Thailand; 2 Institute for Food Quality and Food Safety, University of Veterinary Medicine Hannover (TiHo), 30173 Hannover, Germany; nils.grabowski@tiho-hannover.de (N.T.G.); 3 Faculty of Veterinary Medicine, Mahanakorn University of Technology (MUT), Bangkok 10530, Thailand; rachakris@mut.ac.th (R.L.); tassanee@mut.ac.th (T.T.); 4 Department of Animal Production Technology and Fisheries, School of Agricultural Technology, King Mongkut’s Institute of Technology Ladkrabang (KMITL), Bangkok 10520, Thailand; achara.lu@kmitl.ac.th (A.L.)

**Keywords:** watermeal (*Wolffia globosa*), production performance, optimal level, two-spotted cricket

## Abstract

Watermeal (*Wolffia globosa*) is an emerging sustainable feed ingredient, valued for its rapid growth, high protein content, and rich nutrient profile, offering great potential to improve the efficiency and sustainability of edible cricket production systems through efficient resource use and reduced environmental impact. This study aimed to evaluate the effects of replacing commercial cricket feed with dried watermeal and to determine the optimal substitution level for the production performance of two-spotted crickets (*Gryllus bimaculatus*). Seven dietary treatments were tested, namely 0% (control; T1), 10% (T2), 25% (T3), 50% (T4), 75% (T5), 90% (T6), and 100% (T7). Crickets were reared for four weeks in an open system with 6 replicates per treatment, with each containing 120 nymphs. Body weight, feed intake, and survival rate were recorded weekly, and a production index was calculated at the end of the trial. Results showed average growth rates ranging from 7.40 to 28.20 mg/day, feed conversion ratios (FCR) between 1.03 and 1.68, survival rates of 29.28% to 69.73%, and production index values from 24.00 to 128.30. A significant decline in production efficiency (*p* < 0.05) was observed at substitution levels of 75% and above, with the 100% watermeal group showing the lowest production efficiency (*p* < 0.05), likely due to nutrient imbalances and indigestible compounds. Segmented regression analysis identified 36.7% as the optimal level of watermeal supplementation. In terms of body composition, crickets fed diets containing up to 50% watermeal (10%, 25%, and 50%) showed crude protein, crude fat, crude fiber, and ash contents comparable to those fed the commercial diet. This finding suggests that dried watermeal can replace up to 50% of the commercial cricket feed without negatively affecting growth performance, survival, or nutritional quality, with 36.7% identified as the optimal replacement level for maximizing production efficiency.

## Simple Summary

Watermeal is a high-protein plant, making it a suitable supplementary feed for crickets. In this study, dried watermeal was substituted into commercial feed at levels of 0% (commercial feed only), 10%, 25%, 50%, 75%, 90%, and 100% (watermeal only) over a 28-day experimental period. The results showed that production performance, survival rate, production index, and nutrient content in cricket bodies were acceptable at substitution levels of 10%, 25%, and 50%, while levels of 75%, 90%, and 100% resulted in poorer outcomes. The optimal substitution level of dried watermeal was determined to be 36.7%.

## 1. Introduction

Food production within the framework of a circular economy is crucial for ensuring sustainability and food security. This is especially relevant today, as resources for production are becoming increasingly limited and the environmental impact of food systems continues to grow. Applying circular economy principles to food production is also essential for sustaining life in extraterrestrial environments, where production resources are extremely constrained. Insect production contributes to the circular economy by transforming waste or byproducts into value-added products [1,2]. Cricket farming within a circular economy framework is centered on the production of insects as a sustainable alternative protein source to conventional livestock, such as cattle, pigs, and poultry. Insects offer clear advantages in terms of feed conversion efficiency and produce significantly less environmental waste. One of the main byproducts of cricket farming is cricket frass (excrement), which, according to earlier reports [3], contains essential nutrients, such as nitrogen (2.3–2.6%), phosphorus (1.6–2.0%), and potassium (1.8–2.3%). These nutrient levels make cricket frass suitable for use as an organic fertilizer in crop cultivation. However, growing crops to feed crickets still requires the cultivation of various plants, which demands land and time for production. Conversely, using cricket frass as a source of nitrogen and phosphorus to fertilize watermeal (*Wolffia globosa*) is highly promising and further enhances circularity within a circular economy framework.

The type of feed used in cricket farming is a key environmental factor that influences both population growth and the nutritional content of crickets [4,5]. To ensure affordability, farmers need feed that is inexpensive, accessible, and sustainable [6]. Agro-byproducts and weeds have been proposed as promising alternative materials for formulating cricket feed [5]. Watermeal (*Wolffia globosa*) is a promising alternative plant with the potential to serve both as a protein substitute for soybean meal and as an energy source due to its starch content. Cultivating watermeal under controlled conditions—with the addition of nitrogen and phosphorus to the water—significantly enhances its protein content compared to naturally grown sources [7]. Although environmental and cultivation conditions markedly affect the nutritional and biochemical composition of *W. globosa*, the species consistently demonstrates a high nutritional value, underscoring its potential as a sustainable and nutrient-rich biomaterial for functional food applications [8].

*Wolffia globosa*, a fast-growing aquatic plant native to Southeast Asia, plays a valuable role in advancing circular systems in cricket farming. With its exceptional protein content (26.7% dry weight), high carbohydrate content (48.3% dry weight), balanced amino acid profile, and rich micronutrient composition [9], *W. globosa* is a highly promising alternative feed ingredient for crickets. What makes *W. globosa* particularly relevant to circular economy models is its ability to thrive on nutrient-rich wastewater or agricultural effluents, absorbing excess nitrogen and phosphorus while producing harvestable biomass. According to previous reviews [10], duckweed species, including *W. globosa*, are capable of removing 70–80% of nitrogen and phosphorus from secondary-treated sewage effluent within seven days. Specifically, *W. globosa* exhibits nutrient assimilation rates of 2–6 g N/kg fresh mass/day and 1.6–4.9 g NH_4_^+^/kg/day, demonstrating its high efficiency in nutrient uptake. This dual function—bioremediation and feed production—closes nutrient loops and transforms waste streams into high-value resources. Moreover, *W. globosa* can be cultivated locally with minimal land, energy, and chemical inputs, reducing the ecological footprint associated with imported or land-intensive feed ingredients, such as soybean or fishmeal. Integrating *W. globosa* into cricket farming systems supports circular agriculture by improving feed sustainability, promoting waste valorization, and enabling low-input, high-output protein production. Therefore, using watermeal as feed for crickets is highly feasible. Overall, integrating watermeal cultivation with cricket farming can establish a circular economy-based production system that promotes sustainability and enhances future food security. Regarding the use of *W. globosa* for insect feeding, only one publication in Thai [11] has reported on its dietary supplementation in a commercial diet for house crickets (*Acheta domesticus*). The study found that a 25% inclusion of watermeal resulted in body weights comparable to those of crickets fed a farmer-formulated diet, but lower than those of crickets fed either the commercial diet alone or the commercial diet supplemented with 25% of a mixture containing watermeal, durian peel, and durian seed. To explore the applicability of watermeal in cricket farming, the current study aims to investigate the effects of replacing commercial cricket feed with dried watermeal (*W. globosa*) as a protein source, and to determine the optimal replacement level for improving the production performance of two-spotted crickets.

## 2. Materials and Methods

### 2.1. Rearing and Management of Experimental Crickets

To determine the effects and optimal level of dietary watermeal (*W. globosa*) supplementation on the production performance of two-spotted crickets (*G. bimaculatus*), the crickets were reared at a farm facility of the Animal Science Division, Department of Animal Production Technology and Fisheries, School of Agricultural Technology, King Mongkut’s Institute of Technology Ladkrabang, Thailand. Prior to rearing, a quaternary ammonium compound was used to sanitize the equipment and rearing area. Eggs of two-spotted crickets were purchased from a commercial cricket breeder in Thailand (Somjainuk Cricket Farm, Nakhon Sawan City, Thailand). The eggs were incubated at an ambient temperature of 29.24 ± 0.60 °C and relative humidity of 62.20 ± 9.16% for 7 to 10 days for hatching. The hatched nymphs were reared under mass-rearing conditions as described in previous reports [12,13]. Parajulee et al. [12] reported a mass-rearing yield of approximately 6000 house crickets (≈3000 g) per unit. Both the study by Mitchaothai et al. [13] and the current study achieved a comparable yield—at least 3000 g of field crickets per crate—meeting the criteria for mass rearing. Approximately five weeks after hatching, cricket eggs laid in a clean, moistened mixture of coconut husk fiber and dust were collected for incubation. The eggs were incubated at an ambient temperature of 29.89 ± 0.74 °C and relative humidity of 57.80 ± 10.35% for 7 to 8 days for hatching. In total, 5040 newly hatched nymphs (<1 day old) were randomly selected for use in the current study. A total of 120 nymphs were reared per box, with 6 replicates per treatment. The nymphs were maintained in an open room at an ambient temperature of 29.77 ± 0.76 °C and a relative humidity of 59.81 ± 8.24% until approximately 90% or more of the crickets had reached the adult stage. Cricket rearing and management procedures followed earlier reports [13,14], with significant adaptations for the smaller rearing boxes and some of the equipment employed. Briefly, plastic boxes measuring 24.7 × 35.7 × 32.3 cm (width × length × height) were used for rearing the experimental crickets. Small egg carton trays (19 cm × 22 cm × 4.7 cm) were continuously arranged in a zigzag pattern and stacked in each rearing box to a height of 3–5 layers. Drinking water was supplied daily using moistened, clean gauze placed in a small plastic tray (9 cm × 15 cm × 2.5 cm) with a rough surface. A small sweeper was used weekly to clean the rearing boxes and to collect frass and debris from the floor. Each rearing box was covered with a nylon mesh to allow ventilation, protect against flies, and prevent access by natural predators (e.g., geckos).

### 2.2. Experimental Diet

Experimental diets were prepared by incorporating dried watermeal into a commercial cricket feed (in fine powder form) at inclusion rates of 0% (T1), 10% (T2), 25% (T3), 50% (T4), 75% (T5), 90% (T6), and 100% (T7) on an as-fed basis (Table 1). The commercial feed (Betagro 260^®^, Betagro Public Company Limited, Lop Buri City, Thailand) contained 21.72% crude protein (CP), 3.33% crude fat, and 3928.85 kcal/kg of gross energy (Table 1). The dried watermeal (*W. globosa*) used in the experimental diets contained 23.24% CP, 0.97% crude fat, and 3926.60 kcal/kg of gross energy (Table 1). The diets were offered ad libitum in plastic trays (9 cm × 15 cm × 2.5 cm) with a rough surface. Unconsumed feed was replaced every 2–3 days during the first two weeks and every 1–2 days during the second two weeks of the study. Bulk density was determined by filling a glass cube of known volume with each diet until slightly overfilled. Excess feed was leveled off using a plastic ruler without compressing the contents. The weight of the filled cube was recorded, and bulk density was calculated in g/cm^3^. Each value represents the average of three independent measurements.

### 2.3. Growth Performance, Feed Efficiency, Survival Rate, and Production Index

Growth performance and feed efficiency were measured weekly on days 7, 14, 21, and 28. For each rearing box, crickets were hand-counted and weighed using a digital balance (Sartorius^®^, Sartorius AG, Göttingen, Germany) to determine body weight, body weight gain, and average daily gain (ADG) per cricket. Feed intake per replicate was recorded weekly, and the feed conversion ratio (FCR) was calculated by dividing total feed intake by total weight gain. Survival rate (%) was assessed at the end of each experimental week. On day 28, male and female crickets were identified based on the presence or absence of an ovipositor, and the sex ratio was subsequently calculated. The production index (PI) was calculated weekly using an equation based on the European Broiler Index (EBI) [15], as follows:

EBI = PI = (ADG × Percent survival)/(FCR × 10)

This equation was adapted from its practical use in broiler production, with modifications to express average daily gain (ADG) in milligrams (mg) instead of grams (g).

### 2.4. Sample Collection

All live experimental crickets were fasted for 4 h [16] to allow gut clearance and then harvested on day 28 by transferring the crickets from each rearing box into Ziploc bags. Males and females were collected separately for each box and immediately placed in an ice box for approximately 10–15 min. Subsequently, all cricket samples were frozen and stored at −20 °C until further analysis. Prior to analysis, the frozen crickets were thawed at room temperature, dried at 60 °C for 72 h in a hot-air oven (Model ED 115, Binder GmbH, Tuttlingen, Germany), and then finely ground to 0.5 mm for chemical analysis. Due to the low number of crickets from each replicate in T5, T6, and T7, samples of the same sex were pooled for each experimental treatment, resulting in two samples (one male and one female pooled samples) per treatment. Meanwhile, for T1, T2, T3, and T4, same-sex samples were pooled from two replicates (rearing boxes), yielding six samples (three male and three female pooled samples) per treatment.

### 2.5. Chemical Analysis

Samples of the experimental diets and crickets were analyzed for dry matter (DM), ash, crude protein (CP), crude fat (ether extract, EE), crude fiber (CF), and nitrogen-free extract (NFE), except for the NFE in the cricket sample. The proximate compositions were determined using standard methods recommended by the Association of Official Analytical Chemists (AOAC) [17]. Further details of these analytical procedures are available in an earlier report [13]. Crude protein (CP) content was calculated by multiplying the nitrogen content by a conversion factor of 6.25 for the diet samples, while a factor of 5.0 was used for the cricket samples to account for the high non-protein nitrogen content associated with chitin [13,18].

### 2.6. Statistical Analysis

All production performance data were analyzed using a completely randomized design (CRD) with one-way ANOVA. Significant differences among treatments were determined using Tukey’s multiple range test at a significance level of *p* < 0.05. The segmented regression model of a single breakpoint with 2 simple linear functions was used to describe the slope of the production index (PI) and the level of watermeal supplementation. The segmented regression model was defined as follows:

γ = γ_0_ + γ_1_ × SL + γ_2_ × (SL > xc) × (SL − xc)

where γ is production index, SL is the supplement level of the watermeal (%), γ_0_ is the intercept, γ_1_ is the slope of the line before the breakpoint (knot), γ_2_ is the slope after the breakpoint relative to the slope of the line before the breakpoint, and xc is the value of the breakpoint. The term (SL > xc) is a logical condition that takes the value 1 if true and 0 if false—meaning the expression γ_2_ × (SL − xc) is only included when SL exceeds xc. For the nutrient composition of the crickets, no significant differences were found between male and female crickets fed diets supplemented with watermeal (*W. globosa*) at levels ≤ 50% or >50%. Therefore, the data were regrouped into three categories, namely T1 (0%), T2 to T4 (≤50%) and T5 to T7 (>50%), and a Kruskal–Wallis test was performed to detect statistical differences. All statistical analyses were performed using R software (version 4.4.2) [19]. 

## 3. Results

The supplementation of dried watermeal (*W. globosa*) at varying levels in the commercial cricket feed led to changes in the nutrient composition of the final experimental diets (Table 1). Nutrients present in higher concentrations in watermeal (crude protein, crude fiber, and ash) increased consistently with higher levels of watermeal inclusion. Conversely, nutrients with lower concentrations in watermeal (crude fat, nitrogen-free extract, calcium, and phosphorus) decreased consistently as the level of supplementation increased. The crude protein to NFE ratio increased consistently and proportionally with higher levels of watermeal supplementation, as the protein content increased while the NFE content decreased. However, because the gross energy (GE) content of watermeal was similar to that of the commercial diet, only minor fluctuations in GE content were observed among the different experimental diets. The bulk density of the experimental diets consistently decreased with increasing levels of watermeal supplementation.

The experiment began with 5040 newly hatched two-spotted crickets (<1 day old), with an average initial body weight of 1.12 ± 0.03 mg. No significant differences in initial body weight were observed among the experimental treatments.

**Figure 1:**
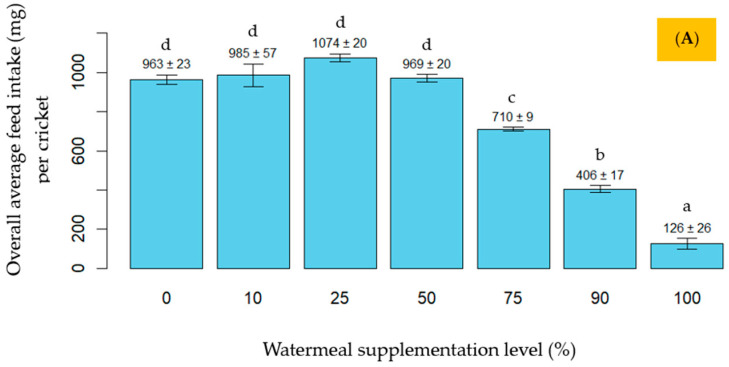

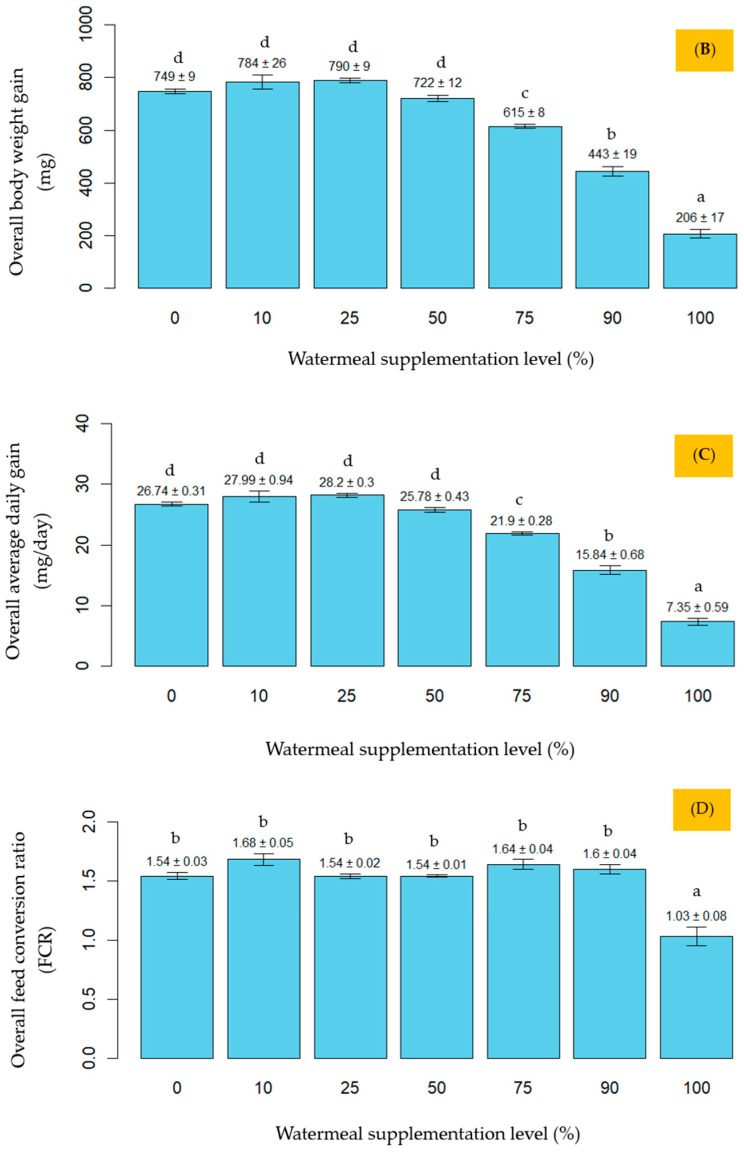
Mean values with error bars for production performance from week 1 to 4: average daily feed intake (**A**), body weight gain (**B**), average daily gain (**C**), and feed conversion ratio (**D**). Different lowercase letters (a–d) above the bars indicate statistically significant differences among treatments (*p* < 0.05).

The influence of different levels of watermeal supplementation on overall 4-week growth performance and feed efficiency is showed in Figure 1A–D. Overall, during weeks 1 to 4 of age, the experimental crickets responded significantly (*p* < 0.05) to increasing levels of watermeal supplementation, depending on both age and supplementation level. Feed intake values increased with age across all seven experimental diets (Supplementary Table S1). When overall feed intake from weeks 1 to 4 was analyzed, no significant differences (*p* > 0.05) were observed among the ≤50% supplementation groups, whereas feed intake declined consistently and significantly (*p* < 0.05) in the >50% supplementation groups as the level of watermeal increased. For body weight gain (BWG) and average daily gain (ADG) during weeks 1 to 4, no significant differences (*p* > 0.05) were observed among the ≤50% supplementation groups. In contrast, both BWG and ADG declined consistently and significantly (*p* < 0.05) in the >50% supplementation groups as the level of watermeal increased. For the feed conversion ratio (FCR), crickets across all seven treatments showed FCR values ranging from 0.16 to 0.47 in week 1 and from 0.70 to 0.95 in week 2, indicating lower feed intake relative to growth during these periods (Supplementary Table S1). For the overall FCR from weeks 1 to 4, crickets fed the 100% watermeal diet (T7) had a significantly lower FCR (*p* < 0.05) compared to those fed 0% (T1), 10% (T2), 25% (T3), 50% (T4), 75% (T5), and 90% (T6) watermeal diets.

**Figure 2:**
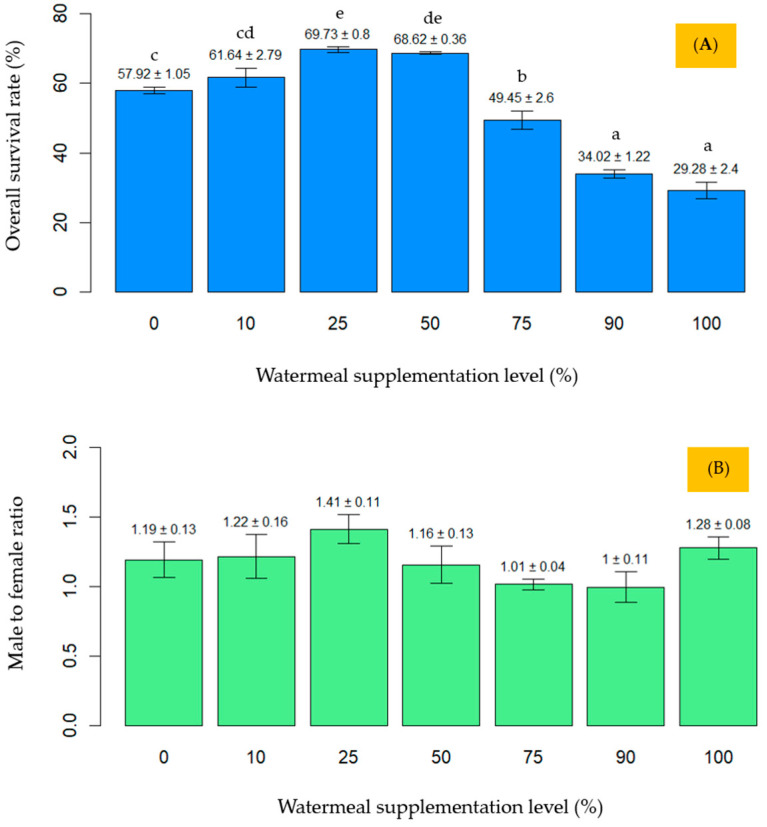
Mean values with error bars for survival rate (**A**) and male to female ratio (**B**) of reared crickets. Different lowercase letters (a–e) above the bars indicate statistically significant differences among treatments (*p* < 0.05).

From the start of the experiment to week 4, the lowest survival rates (*p* < 0.05) were observed in the 90% (T6) and 100% (T7) supplementation treatments, followed by the 75% (T5) group (Figure 2A). These three treatments with >50% supplementation had significantly lower (*p* < 0.05) survival rates compared to the groups with ≤50% supplementation, namely 0% (T1), 10% (T2), 25% (T3), and 50% (T4). The highest survival rate (69.73%) was recorded in the T3 (25%) treatment. No significant differences (*p* > 0.05) in sex ratio were observed among the different protein levels (Figure 2B).

**Figure 3:**
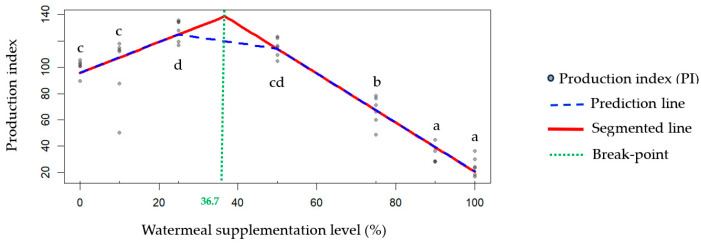
Segmented regression analysis of the production index from week 1 to 4 in experimental crickets, with a breakpoint at 36.7% watermeal supplementation (*p* < 0.001, *n* = 42). Different lowercase letters (a–d) above or below the production index symbol indicate statistically significant differences among treatments (*p* < 0.05).

The trend in the production index closely mirrors that of the survival rate. From the start of the experiment to week 4, the lowest production indices (*p* < 0.05) were observed in the 90% (T6) and 100% (T7) supplementation treatments, followed by the 75% (T5) group. These three treatments with >50% supplementation had significantly lower (*p* < 0.05) production indices compared to the ≤50% supplementation groups, namely 0% (T1), 10% (T2), 25% (T3), and 50% (T4). The highest production index (128.30) was recorded in the T3 (25%) treatment. From the segmented regression analysis (Figure 3), the breakpoint (knot) of the production index was 36.7% watermeal supplementation and the segmented regression model (*p* < 0.001, *n* = 42) was as follows:

γ = 95.69 + (1.18 × SL) + (−3.05) × (SL > 36.7) × (SL − 36.7)

The effect of different levels of watermeal supplementation on the nutritional composition of two-spotted crickets is presented in Table 2. The nutritional values of crickets fed the following diets were compared: (1) 0% (T1) as the control, (2) 10% (T2), 25% (T3), and 50% (T4) grouped as ≤50% supplementation, and (3) 75% (T5), 90% (T6), and 100% (T7) grouped as >50% supplementation. No significant differences (*p* > 0.05) were observed among the three groups for dry matter, crude fiber, and phosphorus. Crickets that received ≤50% supplementation had similar (*p* > 0.05) crude protein, crude fat, ash, calcium, and gross energy contents to those fed the commercial diet (T1). However, compared to the >50% supplementation group, crickets in the control and the ≤50% groups had significantly lower (*p* < 0.05) crude protein, ash, and calcium contents, but significantly higher (*p* < 0.05) crude fat and gross energy contents.

## 4. Discussion

Regarding the optimal crude protein (CP) level in cricket feed, Sorjonen, et al. [20] suggested that crickets could thrive on 22.5% CP, while Kaewtapee et al. [16] reported an optimal level of 21.65% CP. A CP level of 24% appeared to reduce growth performance and feed efficiency [16]. The crude protein (CP) content in the experimental diets of this study ranged from 21.72% to 23.24%, which should be an acceptable and optimal level for rearing two-spotted crickets. For gross energy supply in the diet, Magara, et al. [21] reported values of 3240 and 3563 kcal/kg for rearing *Gryllus madagascariensis*, and 3819 kcal/kg for a reference feed. The experimental diets in the current study contained 3926.60 to 3989.95 kcal/kg, indicating a sufficient energy supply. Regarding bulk density, the dried watermeal (0.231 g/cm^3^) had a bulk density 2.4 times lower than that of commercial cricket feed (0.553 g/cm^3^). Currently, there is no official recommendation for the optimal bulk density of cricket feed. In principle, both excessively low and high bulk densities can negatively affect feed efficiency. Although the present study used dried watermeal to significantly reduce moisture content, the resulting bulk density remained relatively low.

For the feeding trial, the average body weight of newly hatched two-spotted crickets (1.12 ± 0.03 mg) was close to an earlier report of 1.36 mg [13]. The absence of significant differences in initial body weight indicates that the crickets were uniform in size across all groups at the start of the experiment. During weeks 1 and 2 of age (Supplementary Table S1), crickets consumed less feed relative to their body weight gain across all seven experimental treatments, resulting in FCR values below 1.00. In week 2, the FCR ranged from 0.70 to 0.95, which is comparable to the findings of Kaewtapee et al. [16] who reported FCR values between 0.76 and 0.87 for two-spotted crickets reared in central Thailand under similar conditions and ambient temperatures. This low FCR (<1.00) is consistent with the findings of Nakagaki and Defoliart [22], who reported FCR values of 0.923 and 0.949 for crickets fed commercial feed. This efficient feed conversion is likely due to crickets consuming the carcasses of dead individuals, as supported by Vaga et al. [23], who found that nutrients from cricket eggshells, unhatched eggs, and dead crickets can enhance cricket growth. Another key factor is that during the first 7 days of life, insects tend to store more fat than they absorb directly from their feed, primarily due to the conversion of dietary carbohydrates into fat. Changes in the proportion of fatty acids may also influence fat accumulation [24]. In female two-spotted crickets, the lipogenic activity of the fat body, fat body weight, and energy stores in the fat body peak on day 2, in preparation for the development of reproductive functions [25]. From weeks 1 to 4, the FCR ranged from 1.54 to 1.68 (Supplementary Table S1), except in the 100% watermeal group (T7), where the FCR was 1.03 due to low feed intake and limited body weight gain, indicating an imbalance in the nutrient composition of the watermeal. This may be attributed to the presence of indigestible compounds that inhibited growth, along with the increased energy required for excreting excess protein in the form of ammonia and uric acid [26,27]. The FCR range in this study was comparable to the ranges reported in earlier studies, including 1.50–1.81 by Bawa et al. [4] and 1.60–1.72 by Kaewtapee et al. [16]. However, Mitchaothai et al. [13] reported a higher FCR of 2.94 for two-spotted crickets under mass rearing conditions, likely due to greater feed loss from spillage. Thus, measurement of production performance in two-spotted crickets can begin at 7 days of age.

For further insight, the authors classified all seven experimental diets in the current study into two groups based on watermeal supplementation, namely ≤ 50% (T1, T2, T3, and T4) and >50% (T5, T6, and T7). This classification offers a broader perspective and indicates that the growth of the studied crickets was maintained at watermeal supplementation levels of ≤50% but was reduced at levels > 50%. Regarding the protein-to-carbohydrate (P–C) ratio, this study used the protein-to-nitrogen-free extract (P–NFE) ratio as a proxy. However, using NFE may overestimate the available carbohydrate content, as it includes indigestible components. It is presumed that increasing the level of watermeal supplementation led to a higher P–C ratio. When the P–NFE ratios and ADG were considered, diets with >50% watermeal supplementation, which had higher P–NFE ratios, tended to result in a lower ADG. In contrast, crickets receiving ≤50% supplementation showed no such trend. This may be explained by the fact that excessive protein nitrogen can be toxic and stressful to crickets, as its excretion requires substantial energy for conversion into ammonia and uric acid [16,28]. According to earlier reports in fruit flies, lifespan peaked on a diet with a P–C ratio of 1:16, egg production rate peaked at 1:2, and lifetime fecundity (the product of egg production rate and reproductive lifespan) peaked at an intermediate ratio of 1:4 [29]. For *G. sigillatus*, it has been suggested that a P–C diet ratio of 3:1 optimizes life-history traits important for production yield, whereas individuals tend to selectively feed at a more balanced P–C ratio of approximately 1.05:1 [30]. Khempaka et al. [31] reported that house crickets require more dietary energy during the grower phase (21–45 days of age) than in the starter phase (7–20 days of age). They suggested that metabolizable energy (ME) should be determined to gain better insight into nutrient metabolism and to enhance the practicality of cricket diet formulation, as is commonly practiced in swine and poultry nutrition. For survival rate, lower survival was observed in crickets fed diets with >50% watermeal supplementation (higher P–NFE) compared to those fed ≤50% supplementation (lower P–NFE). This may be explained by previous findings that associate longer lifespans in insects with lower P–C ratios [32,33]. Therefore, optimizing the P–C ratio in formulated diets improves feed conversion efficiency, accelerates development, and enhances survival rates, particularly in mass-rearing systems. The ideal P–C balance may vary depending on cricket species, developmental stage, and environmental condition. However, the economic perspective cannot be ignored when applying research findings to practical use.

The lowest feed intake was observed in crickets fed 100% watermeal (T7). A comparable case of feeding 100% brewer’s spent grains (BSGs) was reported by Jucker et al. [27], who observed a very low survival rate (1.5%) and unmeasurable body mass in crickets. In contrast, feeding with 100% watermeal in the present study resulted in a relatively higher survival rate (29.28%) and an average body weight of approximately 207 mg. When BSGs were included at up to 30% in cricket diets, production costs were reduced by up to 29% compared to a commercial diet [34]. Moreover, replacing fishmeal entirely with brewer’s spent yeast or BSGs reduced adult body mass by approximately 16%, although the difference was not statistically significant [35]. These findings are consistent with the current study in terms of production performance and survival rate, suggesting that watermeal can be included at levels up to 50%, assuming no species-specific effects in crickets. Compared to BSGs, watermeal offers better digestibility and a more balanced amino acid profile, making it a preferable ingredient at moderate inclusion levels. However, its seasonal availability and the current lack of large-scale production may limit its broader application. In contrast, BSGs are widely accessible, particularly in tropical regions with expanding brewing industries, and, thus, provide practical advantages within circular agricultural systems. Conversely, feeding with 100% maize distillers grain produced results comparable to those of a commercial chicken diet, which may be attributed to a more balanced nutrient composition for crickets, as reported in the study by Jucker et al. [27].

Crude fiber levels in cricket diets require careful optimization to balance digestive function and nutrient utilization. Appropriate levels can support gut motility without compromising feed efficiency. In contrast, higher fiber levels—particularly those dominated by poorly fermentable components, such as cellulose and lignin—negatively affect growth performance and feed conversion. These findings underscore the importance of controlling both the quantity and quality of dietary fiber when formulating nutritionally adequate and sustainable feeds for crickets. Supporting this, Muzzatti et al. [30] reported that during the first seven days of life, crickets (*Gryllodes sigillatus*) fed high (76%) and medium (45%) cellulose diets exhibited a 174% and 89% increased risk of mortality, respectively, compared to those fed a low (14%) cellulose diet, indicating that high cellulose levels resulted in greater physiological stress.

In the current study, the highest crude fiber content was 11.4% in treatment T7. While this level alone may not cause problems, the type and amount of cellulose and hemicellulose in the watermeal could affect degradation by the gut microbiota of crickets. Thus, further studies should be carried out to elucidate this aspect. In a study on grasshoppers [36], the digestibility of cellulose and hemicellulose varied significantly among species. Cellulose digestibility was higher than that of hemicellulose, and the presence of *Brevibacterium* and *Stenotrophomonas* was significantly correlated with cellulose digestibility. In terms of gut physiology, a study in broilers [37] reported that low bulk density diets may cause gut fill before the animals can consume enough feed to meet their nutrient requirements. In insect science, bulking agents (e.g., cellulose) appear to offer similar effects in insects as they do in vertebrates [38], which suggests that low bulk density diets may cause gut fill before the animals can consume enough feed to meet their nutrient requirements. They may stimulate peristalsis, enhance the texture of solid and semi-solid diets, and reduce the reliance on expensive gelling agents for texture improvement [38]. In livestock production, grinding and pelleting are common methods used to mitigate the effects of bulk density [39]. Further research may be needed to determine the optimal bulking agents and approach for replacing or substituting plant-based ingredients in cricket feed—for example, by supplementing only during a phase with lower protein requirements or grinding leaves into finer particles. Other possible factors and/or interactions may have contributed to the low feed intake observed in T5, T6, and T7; however, this is likely minimal, as crickets in T2, T3, and T4 consumed the diet similarly to those in the control group (T1). Furthermore, two-phase feeding for crickets was reported by Kaewtapee et al. [16] for two-spotted crickets, with phases from 7–18 and 19–35 days of age, and by Khempaka et al. [31] for house crickets, with phases from 7–20 and 21–45 days of age. These two studies aimed to optimize growth performance and feed efficiency in crickets by aligning nutrient provision with phase-specific physiological demands. Consequently, phase feeding could necessitate adjustments in the inclusion levels of watermeal and other nutritive feed components.

Regarding edible cricket production, the industry is expanding and moving toward larger-scale operations. As reported in earlier studies (such as [4,16,21,40,41]), technical parameters, such as the efficiency of conversion of ingested food (ECI), feed conversion ratio (FCR), growth rate or average daily gain (ADG), and survival rate—as well as economic considerations [42]—are commonly used to evaluate the performance of cricket rearing. However, these reports typically focus on only one or two parameters when determining optimal responses to interventions. Therefore, integrating multiple technical parameters would facilitate decision making and provide better insights. In broiler production, standardized indicators, such as the European Broiler Index (EBI) and the European Production Efficiency Factor (EPEF), are widely used to assess production performance. These metrics combine key variables, including average daily weight gain, feed intake, and survival rate. Higher values indicate consistent growth performance and overall flock health, reflecting effective management and environmental conditions [43,44]. The key difference between the EPEF and the EBI is that the EPEF does not require the measurement of initial body weight in broiler chickens. When these two indices are applied to crickets, survival rate and initial body weight are more easily determined under small-scale or laboratory conditions compared to mass-rearing or industrial production. In this study, the EBI was used because the variables required for its calculation can be accurately determined under laboratory-scale conditions. In addition, the production index facilitates ease of use and interpretation for determining the optimal level of dietary watermeal supplementation. To improve applicability, the authors renamed the EBI to the “Production Index (PI)” as a more appropriate term and expressed the measurements in milligrams (mg) instead of grams (g). Based on the results of the segmented regression analysis, the breakpoint (or knot) of the broken-line model was identified at 36.7%, indicating the predicted optimal dose of dietary watermeal supplementation in the commercial feed for two-spotted crickets. This 36.7% value falls within the range of 20–41% for byproduct proportions in barley mash-based diets used for house cricket rearing [45]. However, the predicted value falls within a wide range of watermeal supplementation (between 25% and 50%). Additionally, the PI for the 50% watermeal supplementation did not differ significantly from that of the 0% and 10% supplementation levels. To minimize potential variability associated with higher inclusion rates, future studies should consider testing levels slightly above 25% but substantially below 50%. Based on the available evidence, future research should focus on supplementation levels within the 30–40% range. In addition, when implementing watermeal supplementation for the mass rearing of crickets, reproductive performance (such as fecundity, hatchability, and offspring quality) is a critical factor for achieving successful and sustainable cricket production. However, measuring reproductive performance may have interfered with production outcomes and the crickets’ responses, which represents a limitation of this study in evaluating reproductive traits. Although the eggs obtained from two-spotted crickets in this study were incubated and the resulting nymphs were reared on commercial feed for two subsequent generations without any observed reproductive issues, further research is needed to clarify the effects on reproductive outcomes.

For body cricket nutrients, the crickets offered the watermeal at ≤50% had a similar proportion of nutrient contents with those obtained the commercial cricket feed, while differed from those offered the >50% watermeal supplement. It would be more beneficial to compare the key nutrient contents (CP, crude fat, crude fiber, and ash) of cricket bodies in the present study with those reported in other studies. These nutritive values obtained in this study are consistent with those reported in the previous literature [16,46,47,48]. Earlier studies documented ranges of crude protein (CP) between 42.85–51.03%, crude fat between 21.22–27.95%, crude fiber between 7.27–10.00%, and ash between 2.80–4.11%. These values serve as important benchmarks for evaluating the nutritional quality of two-spotted crickets. The referenced data were derived from various sources, including samples collected in Thailand and other countries, with crickets either raised under controlled conditions or purchased from local markets. This consistency supports the reliability of the current findings and highlights the relatively stable nutrient composition of two-spotted crickets across different rearing environments and geographic locations. It is evident that two-spotted crickets fed diets containing more than 50% watermeal supplementation exhibited increased crude protein (CP) retention but significantly reduced fat retention in their bodies compared to those receiving 50% or less. This may be attributed to the higher P–NFE ratio in the diet, which likely led to reduced feed intake as crickets limited energy intake to avoid the metabolic cost of excreting excess nitrogen, as previously discussed in this section. The resulting lower intake of carbohydrates and fats would directly reduce fat deposition, both through limited dietary fat and through reduced lipogenesis, the process by which carbohydrates are converted to fat in the fat body—a mechanism in most insects that is comparable to that in mammalian tissues [49].

From an overall perspective, dried watermeal can potentially be incorporated into commercial cricket feed at levels of up to 50% for rearing two-spotted crickets. However, based on current findings, the optimal supplementation level is estimated to be approximately 36.7%, as mentioned earlier. Further research is needed to precisely determine the inclusion level that maximizes growth performance, feed efficiency, and survival rate, while also evaluating the economic feasibility for large-scale or industrial cricket production.

## 5. Conclusions

Substituting dried watermeal into the commercial cricket feed for two-spotted crickets at levels of 0%, 10%, 25%, 50%, 75%, 90%, and 100% revealed that inclusion levels up to 50% resulted in comparable growth performance and survival rates to those fed a standard commercial diet. In contrast, crickets receiving more than 50% watermeal supplementation exhibited reduced growth performance and lower survival rates. To evaluate growth performance, feed efficiency, and survivability simultaneously—particularly from an economic perspective—a production index (PI), slightly modified from the European Broiler Index (EBI), was applied. This PI was also used to determine the optimal supplementation level through segmented regression analysis. While some variation in PI was observed among crickets receiving ≤50% watermeal, their values were consistently higher than those of crickets receiving >50% watermeal. These results can be interpreted using the Geometric Framework for Nutrition, commonly applied in insect nutrition studies, as the higher protein-to-nitrogen-free extract (P–NFE) ratios in the latter groups may have led to imbalanced nutrient intake and metabolic inefficiency. Additionally, the key nutrient contents—crude protein (CP), crude fat, crude fiber, and ash—were similar among crickets fed up to 50% watermeal but differed in those fed higher levels, with the exception of crude fiber. These differences are also attributable to variations in the P–NFE ratio and its influence on nutrient metabolism. Based on the PI, the optimal substitution level of dried watermeal in commercial cricket feed was determined to be 36.7%. In addition, the economic feasibility of using watermeal in cricket feed for large-scale or industrial production should be further evaluated.

## Figures and Tables

**Table 1:** Composition and nutrient contents of the experimental feed.

Item	Experimental Treatment						
	T1	T2	T3	T4	T5	T6	T7 *
Ingredients							
Commercial diet (%)	100	90	75	50	25	10	0
Watermeal (%)	0	10	25	50	75	90	100
Analyzed nutrient composition							
Dry matter (%)	87.72	87.99	88.74	89.61	90.89	91.84	93.53
Crude protein (%)	21.72	21.74	21.78	22.02	22.51	22.91	23.24
Crude fat (%)	3.33	3.28	3.03	2.95	2.39	2.17	0.97
Crude fiber (%)	3.32	3.60	4.53	6.12	7.40	8.37	11.70
Ash (%)	6.99	7.32	8.23	9.85	11.29	12.46	13.17
Nitrogen-free extract (NFE) (%)	52.35	52.08	51.18	48.67	47.29	45.93	44.44
Calcium (%)	1.01	0.99	0.98	0.89	0.76	0.65	0.64
Phosphorus, (%)	0.94	0.92	0.86	0.74	0.70	0.61	0.57
Crude protein: NFE	1:2.41	1:2.39	1:2.35	1:2.21	1:2.10	1:2.00	1:1.91
Gross energy (kcal/kg)	3928.85	3934.65	3951.20	3950.85	3972.85	3989:95	3926.60
Bulk density (g/cm^3^)	0.553	0.534	0.432	0.329	0.266	0.236	0.231

* Dried form of watermeal (*W. globosa*).

**Table 2:** Nutrient composition of experimental crickets harvested at 28 days of age.

Item	Control (T1); *n* = 6	Watermeal Supplementation		SEM
		≤50% (T2–T4); *n* = 18	>50% (T5–T7); *n* = 6	
Dry matter (%)	92.48	92.71	91.00	0.32
Crude protein (%)	42.33 ^a^	44.41 ^a^	51.40 ^b^	1.14
Crude fat (%)	28.83 ^b^	24.51 ^b^	12.92 ^a^	1.86
Crude fiber (%)	6.21	7.57	8.43	0.35
Ash (%)	3.74 ^a^	4.10 ^a^	5.25 ^b^	0.21
Calcium (%)	0.19 ^a^	0.17 ^a^	0.49 ^b^	0.06
Phosphorus (%)	0.75	0.76	0.88	0.02
Gross energy (kcal/kg)	6066.50 ^b^	5860.34 ^b^	5167.55 ^a^	116.62

^ab^ Different superscript letters in the same row indicate significant statistical differences (*p* < 0.05).

## Data Availability

The original contributions presented in the study are included in the article. Further inquiries can be directed to the corresponding author.

## Supplementary Materials

The following supporting information can be downloaded at: https://www.mdpi.com/article/10.3390/ani15142052/s1, Table S1: Effects of dietary watermeal supplementation on production performance, survival rate, production index, and sex ratio of experimental two-spotted crickets.
