# Supplementary material for: Effect and Optimal Level of Dietary Dried Watermeal (Wolffia globosa) Supplementation on the Production Performance of Two-Spotted Crickets (Gryllus bimaculatus)
Source: Animals (Basel). 2025 Jul 11;15(14):2052. doi: 10.3390/ani15142052 (PMC12291649; doi:10.3390/ani15142052)
Supplement: Supplementary file 1 [file animals-15-02052-s001.zip › animals-3692002-supplementary.pdf]

Table S1. Effects of dietary watermeal supplementation on production performance, survival rate, production index, and sex ratio of experimental two-spotted crickets.

Item	Experimental group							SEM	F
	T1	T2	T3	T4	T5	T6	T7		($dF_{6, 35}$)
Average feed intake per cricket (mg/cricket)									
Week 1	4.16^{ab}	5.69^{ab}	5.53^{ab}	7.20^{b}	4.04^{ab}	4.53^{ab}	1.36^{a}	0.43	2.19
Week 2	78.29^{c}	102.09^{d}	101.13^{d}	96.06^{d}	80.45^{c}	56.60^{b}	17.72^{a}	4.31	206.8
Week 3	280.26^{c}	396.85^{e}	373.09^{e}	321.38^{d}	264.83^{c}	148.83^{b}	43.07^{a}	18.43	240.68
Week 4	599.81^{e}	480.28^{d}	595.08^{e}	544.73^{de}	361.02^{c}	196.46^{b}	65.16^{a}	30.2	68.52
Week 1-4	962.52^{d}	984.91^{d}	1,074.06^{d}	969.37^{d}	709.96^{c}	405.67^{b}	126.20^{a}	52.5	157.12
Weight gain (mg)									
Week 1	12.62^{b}	15.72^{d}	15.82^{d}	15.43^{d}	14.76^{cd}	13.76^{bc}	8.62^{a}	0.38	299.78
Week 2	84.38^{c}	120.66^{f}	112.87^{ef}	101.90^{de}	91.92^{cd}	64.64^{b}	22.71^{a}	4.91	233.11
Week 3	227.77^{cd}	305.93^{e}	294.48^{e}	257.82^{d}	205.87^{c}	105.37^{b}	34.11^{a}	14.78	239.31
Week 4	277.25^{c}	184.39^{b}	257.75^{c}	238.98^{bc}	82.37^{a}	46.63^{a}	52.48^{a}	16.07	84.36
Week 1-4	748.67^{d}	783.82^{d}	789.54^{d}	721.72^{d}	614.56^{c}	443.45^{b}	205.79^{a}	31.98	196.12
Average daily gain (mg/day)									
Week 1	1.96^{b}	2.39^{cd}	2.41^{d}	2.34^{cd}	2.25^{c}	2.09^{b}	1.38^{a}	0.05	112.69
Week 2	12.86^{c}	17.63^{f}	16.40^{ef}	14.91^{de}	13.73^{cd}	10.52^{b}	4.54^{a}	0.64	148.38
Week 3	36.21^{cd}	47.33^{e}	46.76^{e}	40.45^{d}	35.66^{c}	21.66^{b}	8.72^{a}	2.06	199.51
Week 4	55.86^{d}	44.63^{c}	47.24^{c}	45.40^{c}	36.16^{b}	29.08^{b}	15.97^{a}	1.99	46.3
Week 1-4	26.74^{d}	27.99^{d}	28.20^{d}	25.78^{d}	21.95^{c}	15.84^{b}	7.40^{a}	1.14	196.19
Feed conversion ratio (FCR)									
Week 1	0.33	0.36	0.3	0.47	0.25	0.27	0.16	0.03	1.38
Week 2	0.93^{c}	0.85^{b}	0.90^{bc}	0.95^{c}	0.88^{bc}	0.88^{bc}	0.70^{a}	0.01	25.33
Week 3	1.23^{a}	1.30^{ab}	1.27^{ab}	1.25^{ab}	1.29^{ab}	1.42^{b}	1.27^{ab}	0.02	2.58
Week 4	2.17^{a}	2.94^{ab}	2.35^{a}	2.30^{a}	4.10^{bc}	4.93^{c}	1.64^{a}	0.2	14.3
Week 1-4	1.54^{b}	1.68^{b}	1.53^{b}	1.54^{b}	1.64^{b}	1.60^{b}	1.03^{a}	0.03	22.25
Survival rate (%)									
Week 0-1	87.5	89.72	89.72	90.53	89.45	90.42	84.47	0.6	2.14
Week 0-2	79.60^{bc}	86.65^{cd}	87.50^{d}	87.37^{d}	84.03^{cd}	76.12^{b}	59.58^{a}	1.56	37.6
Week 0-3	71.25^{c}	79.98^{d}	78.32^{d}	78.70^{d}	69.98^{c}	56.65^{b}	40.97^{a}	2.16	105.92
Week 0-4	57.92^{c}	61.64^{cd}	69.73^{e}	68.62^{de}	49.45^{b}	34.02^{a}	29.28^{a}	2.46	83.06
Production index									
Week 1	81.88	65.38	115.4	46.33	124.82	131.32	81.25	13.67	0.82
Week 2	110.57^{bc}	182.40^{e}	160.20^{de}	138.30^{cd}	131.62^{c}	91.50^{b}	38.97^{a}	7.26	48.03
Week 3	210.00^{c}	296.14^{e}	290.00^{de}	256.03^{cd}	194.25^{cd}	87.21^{b}	29.00^{a}	15.23	123.94
Week 4	148.10^{d}	102.10^{bc}	144.00^{cd}	137.40^{cd}	48.50^{ab}	25.00^{a}	32.50^{ab}	9.29	33.76
Week 1-4	100.50^{c}	91.03^{c}	128.30^{d}	115.30^{cd}	67.00^{b}	34.10^{a}	24.00^{a}	6.35	22.78
Sex ratio									
Male:Female ratio	1.19	1.22	1.41	1.16	1.02	0.99	1.28	0.05	1.39

^{abcdef} Different superscript letters in the same row indicate significant statistical differences ($p < 0.05$).
